# High-volume evacuation mitigates viral aerosol spread in dental procedures

**DOI:** 10.1038/s41598-023-46430-3

**Published:** 2023-11-03

**Authors:** Rasmus Malmgren, Hanna Välimaa, Lotta Oksanen, Enni Sanmark, Petra Nikuri, Paavo Heikkilä, Jani Hakala, Aleksi Ahola, Simeoni Yli-Urpo, Ville Palomäki, Eija Asmi, Svetlana Sofieva, Antti Rostedt, Sirpa Laitinen, Martin Romantschuk, Tarja Sironen, Nina Atanasova, Susanna Paju, Laura Lahdentausta-Suomalainen

**Affiliations:** 1 Molecular and Integrative Biosciences Research Programme, Faculty of Biological and Environmental Sciences, University of Helsinki, Viikinkaari 9, 00790 Helsinki, Finland; 2 Department of Virology, University of Helsinki, Haartmanninkatu 3, 00014 Helsinki, Finland; 3 Department of Oral and Maxillofacial Diseases, University of Helsinki and Helsinki University Hospital, Haartmanninkatu 1, 00014 Helsinki, Finland; 4 Meilahti Vaccine Research Center MeVac, Department of Infectious Diseases, University of Helsinki and Helsinki University Hospital, Annankatu 32, 00029 Helsinki, Finland; 5 Faculty of Medicine, University of Helsinki, Haartmaninkatu 4, 00014 Helsinki, Finland; 6 Department of Otorhinolaryngology and Phoniatrics – Head and Neck Surgery, University of Helsinki and Helsinki University Hospital, 00029 Helsinki, Finland; 7 Helsinki University Hospital, 00029 Helsinki, Finland; 8 Aerosol Physics Laboratory, Physics Unit, Faculty of Engineering and Natural Sciences, Tampere University, Korkeakoulunkatu 3, 33720 Tampere, Finland; 9 VTT Technical Research Centre of Finland, Visiokatu 4, 33101 Tampere, Finland; 10 Atmospheric Composition Research, Finnish Meteorological Institute, Erik Palménin Aukio 1, 00560 Helsinki, Finland; 11 Occupational Safety, Finnish Institute of Occupational Health, Neulaniementie 4, 70210 Kupio, Finland; 12 Veterinary Biosciences, University of Helsinki, Agnes Sjöberginkatu 2, 00014 Helsinki, Finland

**Keywords:** Policy and public health in microbiology, Virology, Microbiology, Health care, Dentistry, Disease prevention, Health policy, Occupational health

## Abstract

Dental healthcare personnel (DHCP) are subjected to microbe-containing aerosols and splatters in their everyday work. Safer work conditions must be developed to ensure the functioning of the healthcare system. By simulating dental procedures, we aimed to compare the virus-containing aerosol generation of four common dental instruments, and high-volume evacuation (HVE) in their mitigation. Moreover, we combined the detection of infectious viruses with RT-qPCR to form a fuller view of virus-containing aerosol spread in dental procedures. The air–water syringe produced the highest number of aerosols. HVE greatly reduced aerosol concentrations during procedures. The air–water syringe spread infectious virus-containing aerosols throughout the room, while other instruments only did so to close proximity. Additionally, infectious viruses were detected on the face shields of DHCP. Virus genomes were detected throughout the room with all instruments, indicating that more resilient viruses might remain infectious and pose a health hazard. HVE reduced the spread of both infectious viruses and viral genomes, however, it did not fully prevent them. We recommend meticulous use of HVE, a well-fitting mask and face shields in dental procedures. We advise particular caution when operating with the air–water syringe. Due to limited repetitions, this study should be considered a proof-of-concept report.

## Introduction

Dentists are subjected to saliva and blood-containing aerosols and splatters formed by aerosol-generating instruments on a daily basis^1^. Human saliva contains a multitude of microorganisms, including respiratory viruses such as corona and influenza viruses^2,3^. These viruses are known to spread through aerosol and droplet transmission^4^, causing a potential risk of infection for dental healthcare personnel (DHCP) during dental procedures.

Use of dental instruments, such as the ultrasonic scaler, the air–water syringe, and the air turbine handpiece, are considered aerosol-generating procedures^5–7^. Aerosols and splatters are not only formed by these instruments but also when patients talk and breathe^1,8^. Aerosols can persist in the air for extended periods of time and disperse over long distances, posing in the healthcare setting a potential risk to staff and subsequent patients^9^. Source control using masks or respirators is not possible during dental procedures, and thus, other methods, such as mouth disinfectants, rubber dams, and four-handed dentistry employing a high-volume evacuator, have been used to reduce infection risk during the COVID-19 pandemic^5,6,10^.

With the added threat posed by SARS-CoV-2, routine dental treatments were postponed at the beginning of the pandemic and restrictions were placed on aerosol-generating procedures^10^. These safety measures were introduced quickly out of necessity. However, a well-informed consensus on virus-containing aerosol generation during dental procedures and the infection risk posed was lacking. With this study, we aimed to measure the virus-containing aerosol production of different dental instruments to evaluate the risk that their use poses to DHCP and patients at the dental clinic. Moreover, we combined viral RNA detection with infectious viruses to get a fuller picture of virus-containing aerosol spread in dental procedures.

To examine these phenomena, we simulated dental procedures using common dental instruments: an air turbine, a high-speed dental handpiece, an ultrasonic scaler, and an air–water syringe. A dental phantom head with simulated saliva secretion served as the patient. Bacteriophage Phi6 was chosen as a surrogate as it has previously been used to simulate the spread of influenza and SARS viruses^11,12^.

## Materials and methods

### Overview

Simulated dental procedures were performed on a dental phantom head by three experienced dentists in a clinical setting following typical patient protocols. Buffer containing 1·10^11^ pfu / ml of Phi6 (Supplement Table 1) was pumped into the phantom head’s mouth to simulate stimulated saliva production of 1.5 ml/min^13^. Virus concentration in the buffer was determined before and after experiments using plaque assay. Additionally, a breathing simulation system was used to simulate the effect of the patient's breath on the spread of aerosols.

Virus-containing aerosol production of an air turbine handpiece, a high-speed dental handpiece, an ultrasonic scaler, and an air–water syringe was investigated in 15-min procedures. All procedures were performed on four incisors of the lower jaw. High-volume evacuator (HVE) was used to mitigate the aerosol production. To evaluate its effect, procedures were carried out with HVE (HVE +) and without it (HVE-). At least one HVE + and two HVE- repetitions were measured for each instrument, excluding the air–water syringe (Supplement Table 2). A CompAir Pro C900-nebulizer (Omron, Japan, Kyoto) was used as a positive control, as it produces large amounts of aerosols compared to dental instruments. Phi6-containing buffer was aerosolized with the nebulizer for 10 min with HVE placed 10 cm from the outlet (Supplement Fig. 1).

Viruses were collected passively using Luria–Bertani agar plates containing host bacteria, Pseudomonas syringae sp. HB10Y (HB10Y plates), and actively, using pumps, with 5 ml Biosamplers (SKC Inc., USA, Eighty Four, PA), Button Aerosol samplers (SKC Inc.), and an Andersen 6-stage cascade impactor. Passive collection reflects how aerosols deposit naturally onto surfaces, indicating potential surface contaminants, while active collections represent breathing in contaminated air. HB10Y plates were also used to collect deposing aerosols after the procedures (after plates) in the air turbine handpiece and nebulizer experiments. Additionally, face shield were swabbed after procedures to evaluate possible face contaminations. The face shield was swabbed with a sterile nitrile gloved finger, which was then pressed gently onto an HB10Y plate. The protocol used had been developed by Oksanen et al.^14^ and it was found to retain Phi6 infectivity better than cotton swabs. The number of infectious viruses was analyzed with plaque assay. Virus plaque assay measures the number of infectious viruses by calculating plaque forming units (pfu) per milliliter, with plaque referring to an empty circular area in the bacterial lawn on a petri dish where bacterial cells are lysed due to viral infection. One plaque is formed by the progeny of the first virus infecting the cell. The total virus concentration, including inactive viruses, was analyzed by measuring genome copy numbers with reverse transcriptase quantitative polymerase chain reaction (RT-qPCR)^15^.

Aerosol particle number, size distribution and concentration were measured with an Optical Particle Sizer (OPS, TSI Inc.) model 3330 and the total aerosol number concentration with a Condensation Particle Counter (CPC, TSI Inc.) model 3007. Aerosol levels were monitored between procedures until they returned to baseline.

The dental clinic layout is presented in Supplement Fig. 2. Extended materials and methods are described in the Supplement.

## Results

### Air turbine handpiece

**Figure 1:**
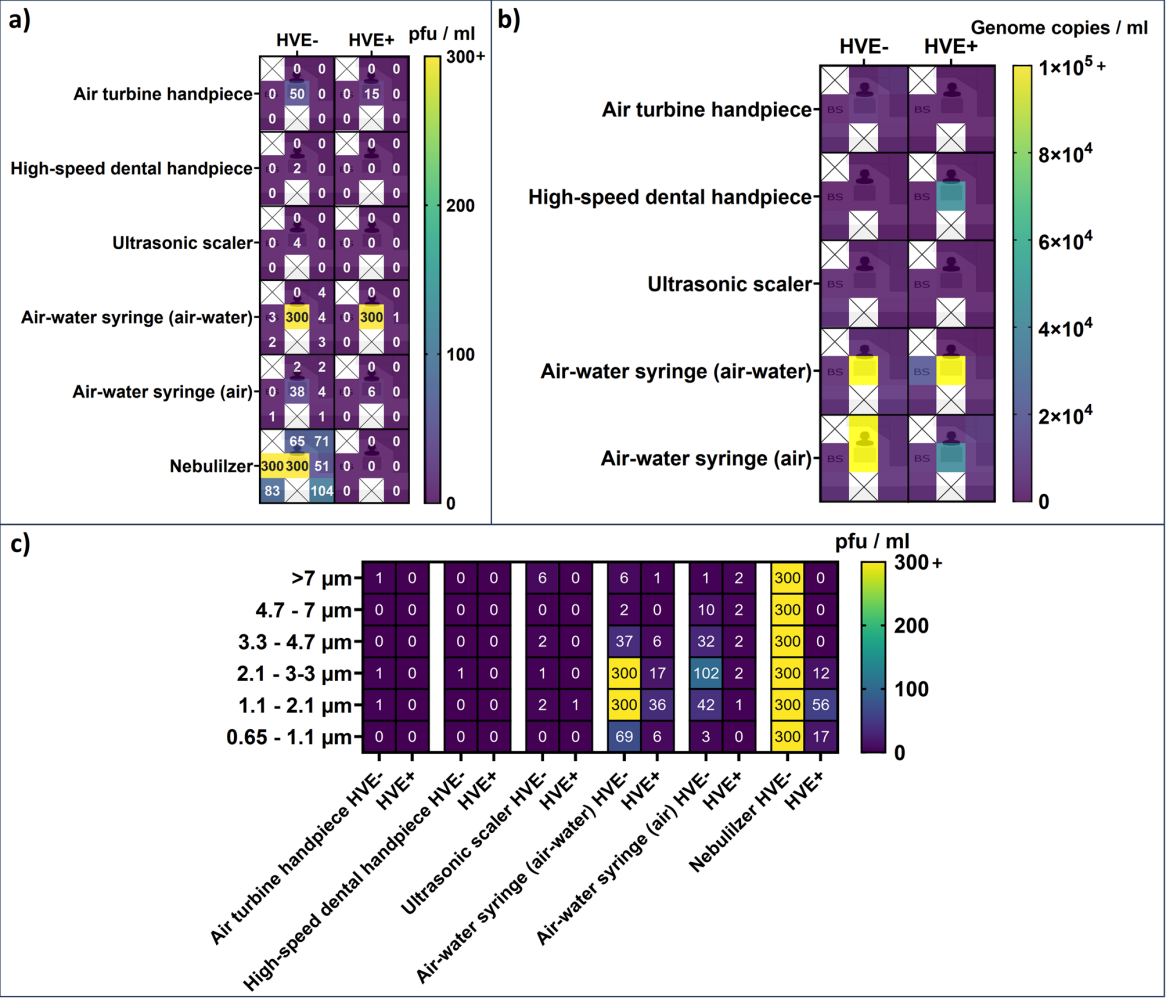
Infectious virus and virus genome detections. (**a**) Infectious virus detections on HB10Y plates and Biosamplers; (**b**) Viral genome copy detections. Middle-left concentration in the clinic images was determined with Biosamplers (BS). (**c**) Infectious viruses detected in Andersen impactor by compartment particle size. Heatmaps were created using GraphPad Prism 10 (https://www.graphpad.com/), layered with the clinic layout created with Smartdraw (https://www.smartdraw.com/).

Infectious viruses were found on plate 1 in HVE + and HVE- procedures, and in the Andersen impactor only in HVE- procedures (Fig. 1). Virus genome copies were detected on all plates in the HVE + procedure. RT-qPCR was unsuccessful for HVE- samples. No viruses were detected with Biosamplers and button samplers (Fig. 1). No infectious viruses were detected on plates opened after the procedures (Table 1).

**Table 1:** Infectious viruses detected on HB10Y after procedures.

Nebulizer after plate	HVE + (pfu)	HVE- (pfu)
Air turbine handpiece plate 1, after procedure	0	0
Air turbine handpiece plate 5, after procedure	0	0
Nebulizer plate 1, after procedure	0	28
Nebulizer plate 5, after procedure	0	23
Nebulizer plate 1, 10 min after procedure	0	7
Nebulizer plate 5, 10 min after procedure	0	5

### High-speed dental handpiece

No infectious viruses were detected using the high-speed dental handpiece in HVE + procedures. However, in HVE- procedures, infectious viruses were detected on plate 1 and one Andersen impactor compartment (2.1–3.3 µm) (Fig. 1).

Virus genome copies were only detected on plate 1 (HVE +) and plate 6.

(HVE-) (Fig. 1).

### Ultrasonic scaler

Infectious viruses were observed on plate 1 and in four Andersen impactor compartments in HVE- procedures. However, in HVE + procedures they were only detected in one Andersen impactor compartment (1.1–2.1 µm) (Fig. 1).

Virus genome copies were detected on all plates in HVE + and HVE- procedures. HVE seems to have reduced the number of detected genome copies significantly (*p* = 0.02), however, the RT-qPCR reliability threshold was not crossed in HVE + samples (Fig. 1).

### Air–water syringe

Significantly (*p* =  < 0.01–0.04) more infectious viruses were detected on HB10Y plates with the air–water and air sprays compared to any other instruments, without HVE. Moreover, significantly more infectious viruses were also detected with the Andersen impactor in HVE + (p = 0.02–0.03) and HVE- (*p* < 0.01–0.03) procedures.

In the air–water syringe procedures, more infectious viruses were detected when using the air–water spray. HVE significantly reduced the number of infectious viruses detected with HB10Y plates when using either the air–water (*p* = 0.05) or air spray (*p* = 0.04). However, infectious virus count decrease with HVE in the Andersen impactor was significant only when using air spray (*p* = 0.03; *p* = 0.09) (Fig. 1). The air–water spray HVE- procedure was the only one where infectious viruses were detected with Button- and Biosamplers; 1 pfu/ml in both Button samplers and 3 pfu/ml in one Biosampler.

More virus genome copies were detected in the air–water and air spray procedures compared to other instruments, with increases ranging up to 730-fold (plate 2). Virus genome copy numbers were significantly higher compared to other instruments for all air spray procedures (*p* =  < 0.01–0.02) and all air–water spray HVE- procedures (*p* =  < 0.01–0.01)  (Fig. 1). When using the air–water spray, virus genome copy numbers were similar in HVE + and HVE- procedures. However, when using the air spray, a 300-fold increase in virus genome copy numbers was recorded on plate 2 in HVE- procedures, compared to HVE + (Fig. 1).

### Aerosol concentrations during dental procedures

**Figure 2:**
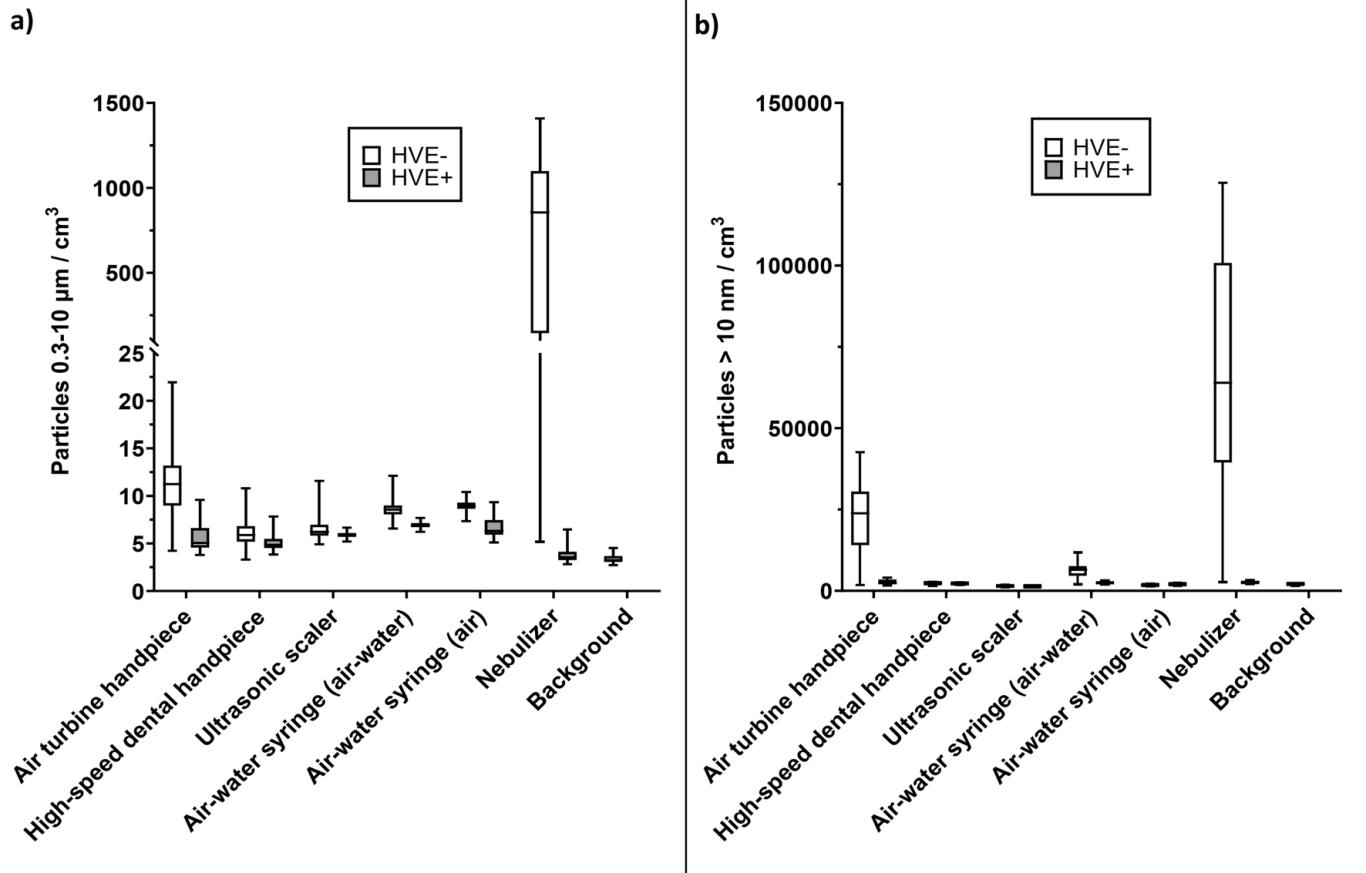
Aerosol concentrations during procedures measured with (**a**) OPS and (**b**) CPC.

Aerosol concentrations remained near baseline in HVE + procedures, whereas in HVE- procedures elevated concentrations were observed. The highest aerosol concentrations were measured with both CPC (45,000/cm^3^, particles > 10 nm) and OPS (21.9/cm^3^, particles 0.3–10 µm) when using the air turbine handpiece. Particle concentration curves from CPC and OPS resembled each other; however, with the high-speed dental handpiece, only particle concentrations in the OPS (i.e. larger particles) were clearly elevated, while measurements with CPC were only slightly elevated. Baseline particle concentrations were 1000–3000 particles/cm^3^ for CPC and 4–6 particles/cm^3^ for OPS, varying slightly between experiments (Fig. 2). HVE significantly (*p* < 0.01) reduced the aerosol generation of all instruments on both ranges.

### Positive control with the nebulizer

Without HVE, aerosol concentrations increased from baseline to almost 50-fold and 250-fold in the CPC and OPS size ranges respectively. Using HVE significantly (*p* < 0.01) reduced the aerosol concentration on both ranges and the aerosol concentration remained near baseline (Fig. 2).

No infectious viruses were detected on HB10Y plates in the HVE + procedure, while in the HVE- procedure, they were detected on all plates. Use of HVE also significantly (*p* < 0.01) reduced the number of infectious viruses detected with the Andersen impactor (Fig. 1). Infectious viruses remained in the air for at least 10 min in the HVE- test, whereas in the HVE + test no viruses were detected on the HB10Y plates after the procedure (Table 1).

### Face shield swabs

Infectious virus plaques were observed on cell culture plates inoculated with face shield swabs after procedures with the ultrasonic scaler and air spray (Table 2).

**Table 2:** Infectious viruses from face shield swabs marked as positive ( +) or negative (-).

Dental procedure	Culture result (HVE +)	Culture result (HVE-)
Air turbine handpiece	–	–
High-speed dental handpiece	–	–
Ultrasonic scaler	–	** + **
Air–water spray	–	–
Air spray	** + **	** + **

## Discussion

Here, by comparing infectious virus and viral genome detections, we provide a new perspective on the spread of viruses in dental procedures. Moreover, this study shows that virus cultivation combined with PCR detection is required to understand the full picture of virus-containing aerosol spread during dental procedures. We demonstrated the importance of HVE in mitigating virus spread and lowering the risk for aerosol exposure of dental healthcare personnel. Air turbine and air–water syringe were found to be associated with a high risk of aerosol generation and virus spread. The use of HVE markedly reduced aerosol concentrations and viral findings. Moreover, we showed that dentists faces are also subjected to infectious virus-containing droplet spills during procedures. Our findings encourage the use of HVE, face shields, and well-fitting masks in dental procedures, not only during pandemics but also in everyday work. However, because this study had only limited repetitions for most instruments, it should be considered a proof-of-concept report, and more studies are needed to bolster these findings.

Previous studies examining the spread of virus-containing aerosols in dental procedures have focused on drill-type instruments^16,17^ and ultrasonic scalers^18,19^. The present study provides data on virus-containing aerosol generation of additional common dental instruments and compares them in the same experimental setup. Moreover, RT-qPCR and infectious virus detection have not been combined before to compare the spread of infectious and inactive viruses in dental procedures. Studying these side by side gives a more complete view on the subject.

In air turbine, high-speed dental handpiece, and ultrasonic scaler procedures, infectious viruses were only detected near the source, indicating that while viral aerosol transmission is possible during these procedures the risk seems to be high mainly at close proximity. Some of the viruses found on deposition plates near the source are of larger droplet spill origin, as visible droplets could be seen on these plates and around them. However, as some viruses were detected with the Andersen impactor situated behind the phantom head in HVE- procedures, both origins should be considered in risk management measures.

In the air–water syringe procedures, more infectious viruses were observed compared to other instruments, especially in the Andersen impactor. The air–water syringe procedures were the only ones where infectious viruses were detected on deposition plates other than next to the source, indicating that it produces infectious virus-containing aerosols. The results from the Andersen impactor are particularly interesting, as infectious viruses were mostly detected from particles smaller than 4.7 µm, with the peak being at 2.1–3.3 µm. This size range is similar to that of SARS-CoV-2 aerosols^20^ and IAV H1N1 aerosols^21^. This size range, which is commonly observed for virus-containing aerosols^4^, seems to apply to the other instruments as well, but because the virus numbers were low in those procedures, more studies are needed to confirm this.

By contrast, viral RNA was detected at all sampling points in all but the high-speed dental handpiece procedures, indicating that aerosols formed during these procedures include viruses, but they are inactivated during aerosolization. This can be due to the extreme physical stress caused by the high rotary speed or the air and water jets of the instruments. The drying of the aerosols also plays a key role in virus inactivation^22^. This effect of drying on the inactivation of viruses is supported by our results with the air–water syringe, where using the air spray reduced the number of infectious viruses detected with all collection methods, compared with using the air–water spray, without reducing the number of detected genome copies. Regarding virus genome analysis, no virus genome was detected in air turbine handpiece HVE- procedures, even though most of the HVE + procedure’s samples crossed the RT-qPCR reliability threshold. We suspect that the collection plates were not properly rinsed during sample collection.

Regarding Phi6 as a survival model for other enveloped viruses, it is important to mention that while it does not perfectly resemble them^23^, it has been found to have similar survivability as SARS or influenza viruses in some respects such as temperature and chemical stress^24,25^. However, because different viruses vary in the loss of infectivity, these results highlight the need for proper protective gear for personnel.

A higher-than-normal virus concentration had to be used in the mock saliva, as infectious viruses would have been impossible to reliably detect otherwise. The RT-qPCR protocol applied also required this, as it has a high reliability threshold of 2⦁10^3^ genome copies / ml^15^. Still, even with a high virus concentration, some of the samples did not reach the reliability threshold and therefore cannot be reported with certainty. This was especially a problem with the ultrasonic scaler. Nevertheless, as it was the only instrument with which most sampling points did not cross the threshold, we can estimate that it is the least dangerous of the investigated instruments in terms of virus-containing aerosol generation.

Our results show that viral aerosol transmission during dental procedures is possible, and thus, appropriate safety measures should be taken. Moreover, with viral genomes detected at many of the sampling points where no infectious viruses were found, it is possible that more resilient viruses, such as non-enveloped viruses^26^, could remain infectious and spread throughout the room, and should therefore be studied in a similar setting. Moreover, bacteria or parts of bacteria^27^, such as endotoxins, spreading in the room could also pose a health hazard^28,29^.

Based on these results, we recommend restrictions on the use of the air–water syringe in worse epidemic situations, as it was found to allow more infectious viruses to spread than any other instrument. We also conclude that the use of a high-speed dental handpiece is safer in terms of aerosol production and virus spread than the air turbine handpiece. Moreover, it can be used as a substitute for the air turbine handpiece during a pandemic or with infectious patients, as has been shown before^17^. This is especially important if a rubber dam or HVE cannot be used.

HVE has previously been shown to reduce aerosol spread during dental procedures^30,31^. Our results support this finding, as HVE was observed to play a key role in mitigating the spread of virus-containing aerosols. This is further supported by the positive control, where HVE was able to almost fully negate the spread of aerosols created with the nebulizer, even though it produces a much higher concentration of aerosols than any of the dental instruments used in this study. HVE also seems to be able to slightly reduce the number of droplets escaping the mouth, as demonstrated by the reduction of viral plaques on the nearest deposition plate during HVE + procedures. However, differences in the work of the dental staff might also be the cause.

Generally, HVE is advised to be positioned as close to the working area as possible, but its effectiveness is also affected by the operator^32^. In the nebulizer test, HVE remained in an optimal position regarding the nebulizer outlet. However, an optimal positioning cannot always be achieved with patients, which can result in peaks in aerosol concentrations, as seen in some of the HVE + procedures (Fig. 2). The aerosols formed from dental instruments also likely have a higher velocity than those produced with the nebulizer, which can allow some particles to pass the HVE. Based on these results, we recommend always using HVE during dental procedures to mitigate aerosol spread. Moreover, a wider HVE should be used when available^33,34^. Training is also required to ensure its proper use and positioning.

As Teichert-Filho et al.^35^ found, splatters formed in dental procedures are found all around the DHCP’s clothes and face. Moreover, in the present study, we were able to detect infectious viruses in the face shields of the DHCP, indicating that without protection their faces would be directly subjected to infective viruses. Based on these results, we recommend that all DHCP in the room wear face shields in addition to well-fitting masks during procedures to prevent splatters from reaching the face. Wearing proper face protection is especially important when using high splatter-causing instruments such as the air–water syringe or the air turbine handpiece.

Although not all procedures gave virus-positive results from shield swabs, they showed that DHCP’s faces are at risk of being exposed to infectious viruses from patient’s saliva and blood. Previously, HVE has been found to not significantly affect the viral genome copy numbers detected on DHCP’s face shields^36^, indicating that viruses found on the shields are most likely of splatter origin. Moreover, the plates directly in front of the mouth almost invariably had viruses on them, indicating that viruses from the mouth spread well via splatters formed during procedures. While it is important to acknowledge that the positioning of DHCPs also plays a key role in avoiding these splatters, a safe position cannot always be taken.

We demonstrated the importance of high-volume evacuation in mitigating viral aerosol spread during dental procedures. We also discovered that most, but not all, viruses are inactivated in the process of aerosolization with dental instruments. We recommend caution when operating with the air–water syringe, as it was found to preserve virus infectivity in aerosolization better than any other instrument. Using a face shield, instead of routine goggles, in addition to a well-fitting mask, should be considered in all dental procedures to decrease exposure to infectious agents. Replacing the surgical mask with a respirator is reasonable when treating patients with confirmed or probable infection transmissible by aerosols or droplets.

### Supplementary Information

Supplementary Information.

## Data Availability

The data that support the findings of this study are available from the corresponding author, R. Malmgren, upon reasonable request.
