# Supplementary material for: High-volume evacuation mitigates viral aerosol spread in dental procedures
Source: Sci Rep. 2023 Nov 3;13:18984. doi: 10.1038/s41598-023-46430-3 (PMC10624893; doi:10.1038/s41598-023-46430-3)
Supplement: Supplementary file 1 — Supplementary Information. [file 41598_2023_46430_MOESM1_ESM.pdf]

Supplement

Experimental setup

A dental phantom head, a plastic head simulating face and cheeks with artificial plastic teeth (Frasaco teeth), was in a fixed 11 o'clock position in a dental chair. The height of the phantom head's mouth from the floor was 120 cm. Mock saliva was pumped into the phantom head's mouth at 1.5 ml/min using an Ecoline model ISM1076A pump (Ismatec, Switzerland, Glattbrugg).

Dental instruments

The procedures were performed using an air turbine handpiece (KaVo, Germany, Biberach), a high-speed dental handpiece (KaVo), an ultrasonic scaler G6-tip (NSK Varios 750), and an air-water syringe. The air-water syringe was used with air-water spray and with air only. The HVE applied was the dental unit's suction (Planmeca Compact i), with saliva evacuation at 80 ml/s.

Breathing simulation

A proportional valve-operated breathing simulation system was connected to the phantom head's nose via tygon tubing. The system provided approximately sinusoidal breathing cycles with 2.5 s of inflow (peak flow rate 24 lpm), 3 s of outflow (peak flow rate 16 lpm), and a break of 1 s. The tubing was attached to the nose, mimicking realistic flow jet directions and flow rates characterized by Gupta et al. [1].

Viruses, microbial strains, growth media and plates

Purified Phi6 was used as a model virus and *Pseudomonas syringae* pv. phaseolicola HB10Y (HB10Y) as its host. Phi6 was purified using the sucrose gradient method described by Bamford et al. [2]. As Phi6 is human-safe, it enables the assessment of viral

aerosol spread during dental procedures without any risk to the staff. Phi6 is a suitable mechanical model for SARS-CoV-2; it is an RNA virus of comparable size to SARS-CoV-2 (~ 80–100 nm), similarly enveloped by a lipid membrane, has spike proteins, and is also preferred in other similar studies, including studies of virus survival in human saliva microdroplets [3]. Both Phi6 and *P. syringae* were originally obtained from Ann Vidaver, University of Nebraska [4]. Agar plates containing HB10Y (HB10Y plate) were prepared by mixing 100 µl of bacteria grown overnight at room temperature (RT, 22°C) in Luria-Bertani-Lennox (LB) broth (Supplement Table 2) with 3 ml of LB soft agar and pouring it on LB agar plates.

Virus collection

HB10Y plates and empty petri dishes were placed throughout the room (Supplement Figure 2) to collect passively deposited aerosols. Plates were open only during the procedures. “After” plates were opened immediately after the procedure for 15 min to monitor the number of viruses that were deposited from the air after the procedures. After sample collection, HB10Y plates were incubated overnight (22°C). The empty petri dishes were washed with 1 ml of HEPES (Supplement Table 3) and analyzed with RT-qPCR.

Two 5 ml Biosamplers (SKC Inc.), two Button samplers (SKC Inc.), and a six-stage Andersen cascade impactor were used to actively collect aerosols. Biosampler 1 was filled with 5 ml of HEPES and Biosampler 2 with 5 ml of LB. A pump created an airflow of 12.5 l/min through the Biosamplers. The positions of the Biosamplers are shown in Supplement Figure 2.

The Andersen impactor was filled with HB10Y plates and was used to collect infectious viruses in aerosol droplets of different size ranges. A pump was adjusted to

create a 28.3 l/min airflow through the collector. The airflows were calibrated using Mass Flowmeter 3063 (TSI Inc., USA, Minnesota City, MN).

The DHCP wore Button samplers on their chest during the procedures, which collected particles of <100 µm. The Button samplers were connected to Gilian 5000 air-sampling pumps (Sensidyne, USA, St. Petersburg, FL) using a 4 l/min airflow. Mixed cellulose ester filters (1.2 µm, SKC Inc.) were used in the Button samplers.

Aerosol measurements

The total aerosol particle number concentration at diameters >10 nm was measured with a Condensation Particle Counter (CPC), model 3007 (TSI Inc.) The CPC flow rate was 0.8 l/min and a 1-s time resolution was used in data logging. The flow rate was validated using a calibrated Gillian flowmeter before and after the measurements and the data were corrected accordingly.

An Optical Particle Sizer (OPS) model 3330 (TSI Inc.), flow rate 1 l/min, was used to measure the aerosol particle number size distribution at the range 0.3-10 µm, divided into 16 channels. Flow rate was verified at laboratory settings prior and after the measurements, similar to the CPC. The OPS was operated at 10-s time resolution. The positions of the aerosol measurement devices are shown in Supplement Figure 2.

RT-qPCR

The RT-qPCR analysis was carried out according to Gregorova et al. [5]. A sample volume of 5 µl was used. Data analysis was done using QuantStudio™ Design & Analysis Software v1.5.2. The method had a detection threshold of $2 \cdot 10^3$ genome copies / ml, which was due to the small sample volume used in the protocol.

Personal protection

While Phi6 has not been reported to be harmful for humans [6], it might still cause irritation or other symptoms if large quantities are inhaled. The virus solution apparently contains small amounts of bacterial endotoxins even after purification [7]. Therefore, the DHCP and scientists wore protective overalls, nitrile gloves, eye protectors, and FFP3 respirators during the procedures. Additionally, DHCP wore face shields.

Room layout

The procedures were carried out at the Hospital District of Helsinki and Uusimaa Oral Diseases Teaching and Dental Care Unit in Ruskeasuo, Helsinki. The room was 15.7 m^2 and featured a Compact i dental unit (Planmeca, Finland, Helsinki). Air was supplied to the room from above the dental unit at a rate of 35 l/s and extracted from the bottom left corner of the room (Supplement Figure 2) at a rate of 44 l/s. All windows and doors were kept closed during the procedures.

Statistical analyses

Statistical analyses were performed using GraphPad Prism 10. Comparisons between procedures were performed using the Mann-Whitney test (two-tailed), as the datasets were small and not normally distributed.

Supplement Table 1. Buffer containing Phi6. Substances were dissolved in Milli-Q water

**Substance**	**Concentration**
K-phosphate	20 mM / l
$MgCl_2$	1 mM / l
1 x purified Phi6, c = $1{\cdot}10^{14}$ pfu / ml (Bamford et al. 1995)	$1{\cdot}10^{11}$ pfu / ml

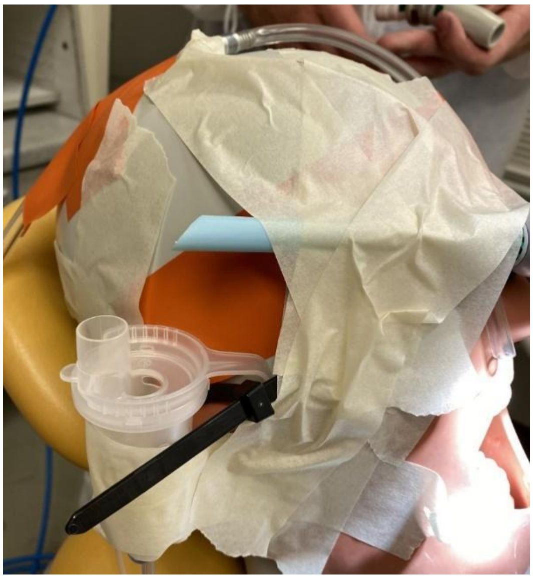

Supplement Figure 1. Nebulizer output and high-volume evacuation positions on the dental phantom head.

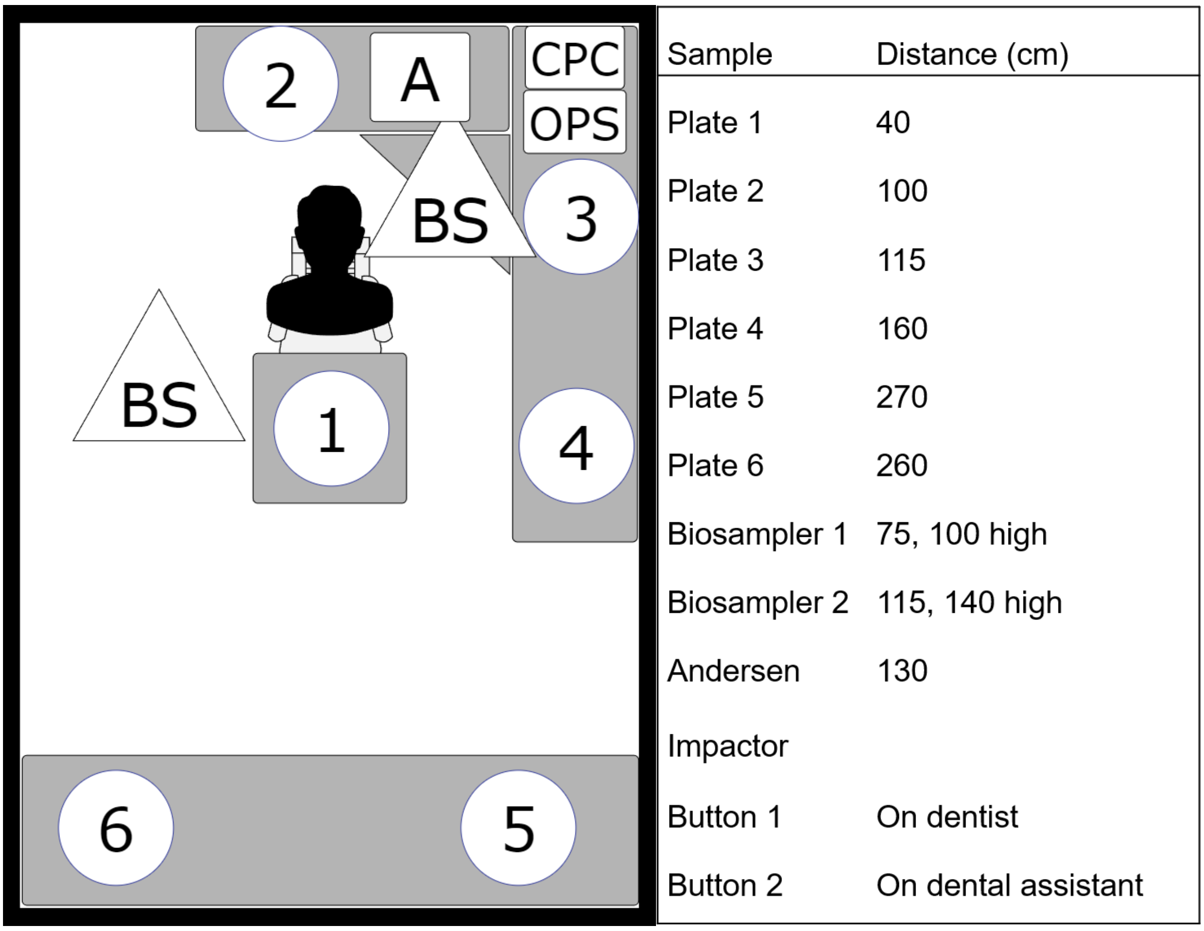

Sample	Distance (cm)
Plate 1	40
Plate 2	100
Plate 3	115
Plate 4	160
Plate 5	270
Plate 6	260
Biosampler 1	75, 100 high
Biosampler 2	115, 140 high
Andersen Impactor	130
Button 1	On dentist
Button 2	On dental assistant

Supplement Figure 2. Dental clinic layout and sampling points. Biosamplers marked with "BS" and Andersen impactor with "A".

Supplement Table 2. Experiment repetitions.

Dental procedure	HVE- repetitions	HVE+ repetitions
Air turbine handpiece	3	2
High-speed dental handpiece	2	1
Ultrasonic scaler	2	1
Air-water spray	1	1
Air spray	1	1

Supplement Table 3. HEPES buffer (20 mM). HEPES was dissolved in Milli-Q water and pH adjusted to 7.2 with KOH. The solution was filter-sterilized using a 0.2 µm filter.

**Substance**	**Concentration**
4-(2-hydroxyethyl)-1-piperazineethanesulfonic acid (HEPES)	4.77 g / l
Potassium hydroxide (KOH)	to adjust pH to 7.2

Supplement References

[1] Gupta, J. K., Lin, C.-H. & Chen, Q. Characterizing exhaled airflow from breathing and talking. *Indoor Air*. **20**(1):31–39 (2010).

[2] Bamford, D. H., Ojala, P. M., Frilander, M., Walin, L. & Bamford, J. K. H. Isolation, purification, and function of assembly intermediates and subviral particles of bacteriophages PRD1 and σ6. *Methods Mol. Biol*. **6**:455–474 (1995).

[3] Fedorenko, A., Grinberg, M., Orevi, T. & Kashtan, N. Survival of the enveloped bacteriophage Phi6 (a surrogate for SARS-CoV-2) in evaporated saliva microdroplets deposited on glass surfaces. *Sci. Rep*. **10**(1):22419 (2020).

[4] Vidaver, A. K., Koski, R. K., Van Etten, J. L. Bacteriophage φ6: a lipid-containing virus of *Pseudomonas phaseolicola*. *J. Virol*. **11**(5):799–805. (1973).

[5] Gregorova, P., Heinonen, M.-M. K. & Sarin, L. P. An improved RT-qPCR method for direct quantification of enveloped RNA viruses. *MethodsX*. **9**:101737 (2022).

[6] Turgeon, N., Toulouse, M.-J., Martel, B., Moineau, S. & Duchaine, C. Comparison of five bacteriophages as models for viral aerosol studies. *Appl. Environ. Microbiol*. 80(14):4242–4250 (2014).

[7] Sanmark, E. *et al*. Safe use of Phi6 in the experimental studies. *Heliyon*, ***9***(2), e13565 (2023).
